# From bottom-up to cell surface proteomics: detergents or no detergents, that is the question

**DOI:** 10.1042/BST20231020

**Published:** 2024-04-26

**Authors:** Zora Brough, Zhiyu Zhao, Franck Duong van Hoa

**Affiliations:** Department of Biochemistry and Molecular Biology, Faculty of Medicine, Life Sciences Institute, University of British Columbia, Vancouver, British Columbia, Canada V6T 1Z3

**Keywords:** membrane mimetics, proteomics, transmembrane proteins

## Abstract

Measuring the expression levels of membrane proteins (MPs) is crucial for understanding cell differentiation and tissue specificity, defining disease characteristics, identifying biomarkers, and developing therapeutics. While bottom-up proteomics addresses the need for accurately surveying the membrane proteome, the lower abundance and hydrophobic nature of MPs pose challenges in sample preparation. As MPs normally reside in the lipid bilayer, conventional extraction methods rely on detergents, introducing here a paradox — detergents prevent aggregation and facilitate protein processing, but themselves become contaminants that interfere with downstream analytical applications. Various detergent removal methods exist to mitigate this issue, including filter-aided sample preparation, SP3, suspension trapping, and membrane mimetics. This review delves into the fundamentals of each strategy, applications, merits, and limitations, providing insights into their effectiveness in MP research.

## Introduction

Membrane proteins (MPs) integral to the lipid bilayer play pivotal roles in diverse cellular functions, influencing signaling, nutrient transport, ion channeling, enzymatic activities and more [1]. Their abundance and activity levels directly contribute to cell and tissue function [2], and thus dysregulation has been linked to neurodegenerative diseases, cancer, and other conditions like hypertension and muscular dystrophy [3–6]. As such, understanding MPs’ expression in cells and tissues is crucial for insights into normal and diseased states. Additionally, plasma MPs (PMPs) are strategically located at the cell surface and targeted by nearly half of therapeutic drugs, despite constituting a relatively tiny fraction of the overall proteome [7,8]. In essence, precise knowledge and quantification of the membrane proteome are vital for understanding disease progression, identifying biomarkers, and advancing drug development. With this in mind, bottom-up proteomic research opens up the pathway to address these critical aspects as the method directly surveys the cell protein content.

Bottom-up proteomics consists of key major steps: sample preparation, liquid chromatography-tandem mass spectrometry (LC–MS/MS), and data analysis [9]. In this well-developed workflow, sample preparation remains the major bottleneck due to the complexity of the biological samples and the multi-stage processes associated with it. Sample preparation typically involves lysing cells, homogenizing, solubilizing and unfolding proteins, reducing disulfide bonds, alkylating sulfhydryl groups, and fragmenting them into peptides for efficient LC separation and MS-detection [9]. This complexity is exacerbated for MPs that naturally exist in lower abundance compared with soluble proteins [10]. Frequently, MPs derived-peptides are less efficiently detected in bottom-up proteomics, let alone robust quantification. MP isolation, and depletion of soluble proteins (as it is not possible to eliminate) that easily overshadow MP-derived peptides, are essential steps. Various enrichment strategies have been developed over the years, including ultracentrifugation, chemical labeling, adhesion- and affinity-based capture, as well as phase and two-phase separation methods among others. Previous reviews have provided a comprehensive description and evaluation of these methods [11–14]. Despite these advances, it is important to note that MPs still need to be extracted from their lipid bilayer environment before analysis, further complicating matters.

Integral MPs feature an extensive hydrophobic stretch, each typically 20–25 amino acids long [8]. This characteristic causes favorability to the lipid bilayer but aggregation in aqueous solutions [10]. Thus, MPs are generally extracted and kept soluble with amphiphiles, most often detergents. These detergents, when in quantities well above their critical micelle concentration (CMC), effectively disrupt the lipid bilayer to extract MPs [15]. Ideally, they also keep MPs in a monodisperse state, free of lipids and other impurities including insoluble aggregates [15]. Strong ionic detergents such as SDS and RIPA (an SDS-based detergent mixture widely used for tissue solubilization) have long been employed in bottom-up proteomics [16]. However, strong ionic detergents bring a multitude of problems with them. Firstly, they diminish the catalytic activity of proteases and lead to insufficient digestion [17]. Secondly, they disturb the chemistry of the reversed-phase (RP) LC, deteriorate the columns [18], modify peptide ionization, and suppress signals during MS [19]. Milder detergent alternatives, such as bile acid SDC and glycoside-based DDM, offer better compatibility with bottom-up proteomic, but are less efficient at membrane solubilization and at disrupting intra- and inter-protein interactions [20,21]. Other mild detergents containing polyethylene glycol (e.g. Triton X-100, Tween 20, or NP-40) are retained and eluted throughout the LC column, requiring removal similar to strong ionic detergents [22]. Chaotropic urea and guanidine increase the solubility of MPs by disrupting water bonding, but their variable solubilization efficiency introduces bias and therefore are not suitable for quantifying MPs [15]. Other reagents such as acetonitrile, ammonium bicarbonate and RapiGest have been effectively employed in digestion protocols, however, they have only shown effectiveness with minute quantities of protein [23]. Thus, strong ionic detergents remain the standard in the field, provided they are carefully eliminated downstream. Unfortunately, in membrane proteomic, detergent removal rapidly leads to protein aggregation and precipitation, creating a dilemma between the need for detergents and their subsequent removal [24]. These issues together contribute to an overall limited understanding of the membrane proteome compared with soluble proteins, despite its crucial importance [25].

Given the complex relationship between detergents and proteins, optimizing the detergent removal process has become an essential preparative step in bottom-up proteomics. This review highlights the recent method developments that are tackling this issue, focusing on the methods that best preserve the cell surface membrane proteome. We cover filter-aided sample preparation (FASP) and modifications, single-pot solid-phase-enhanced sample preparation (SP3), SP3-solvent precipitation (SP4), suspension trapping (S-Trap) and amphiphilic membrane mimetics. The FASP, SP3, SP4 and S-Trap methods typically remove detergents after MPs preparation and purification. In contrast, membrane mimetics enable MP isolation immediately after solubilization, which minimizes detergent exposure, reduces the risk of protein aggregation, and overall simplifies MS-sample preparation [26]. They also keep MP in a folded and water-soluble state usable in applications beyond protein identification [26]. As such, the recent development of membrane mimetics is included in this review.

## Filter-aided sample preparation

**Figure 1.**
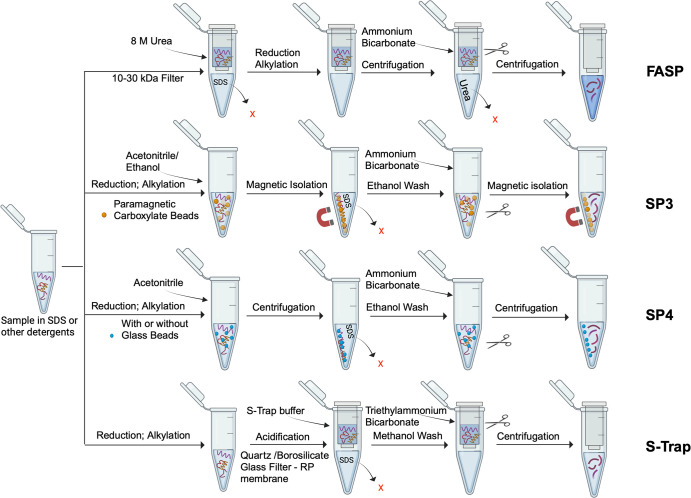
Detergent removal workflows for methods discussed in this review. In these methods, membrane protein enrichment is done prior to detergent removal. FASP exchanges detergent to urea using 10–30 kDa cut-off filters. Samples are reduced, alkylated and digested on the same filter. SP3 depletes detergent after protein binding to paramagnetic carboxylate beads (CMMBs) while SP4 depletes detergent following protein precipitation with organic solvents with or without glass beads. S-Trap captures protein aggregates in S-buffer (phosphoric acid/methanol) over borosilicate glass filters placed over a reverse-phase membrane to capture digested peptides. In SP3, SP4 and S-Trap, samples are reduced and alkylated prior to detergent removal.

Introduced by Manza et al. [27] and refined by Wisniewski et al. [28], FASP is an ultrafiltration-based approach using a regenerated cellulose membrane with a 10–30 kDa cut-off range. This filtration captures SDS-denatured proteins while allowing contaminants to pass through [27,28]. While SDS is the preferred solubilization detergent for FASP, successful depletion of RapiGest and NP-40 detergents has also been achieved [29]. Once the proteins bound the membrane, detergent exchange is performed with a high concentration of urea [30,31]. This serves multiple purposes: removing small contaminants like lipids, carbohydrates, salts, and nucleic acid, while replacing SDS to ensure that proteins remain in an unfolded state amenable to protease digestion. After dilution of urea, the processes of reduction, alkylation, and digestion take place on the same filter. The ultrafiltration system also enables the separation of digested peptides from undigested proteins (Figure 1) [30,31]. This method is very effective on soluble proteins, but some uncertainties remain regarding its compatibility with MPs that may aggregate onto the cellulose surface upon detergent removal, leading to diminished overall protease access and incomplete digestion.

Mann's research team tested FASP on MP identification starting with density-gradient fractionations as a membrane enrichment method [32]. They identified over a thousand MPs in a single LC–MS/MS run using hippocampal mouse tissue as a model. Among the proteins identified, 26% were annotated as PMPs [32]. Yu et al. [33] integrated FASP with gel-electrophoresis fractionation and identified over 770 MPs in a leukemia cell line. In Raimondo et al. [34], 721 proteins were identified from human renal carcinoma, with 53% being membrane-bound. These authors identified differentially expressed MPs, establishing those as novel biomarkers for renal cancer. More recently, Li et al. combined a cell surface labeling procedure (CSL; intact cells are incubated with sulfo-NHS-LC-biotin and labeled protein are isolated with streptavidin beads) with fractionation and FASP to heighten the sensitivity for PMPs [35,36]. In their study on HeLa cells, a total of 4510 proteins were identified, of which ∼46% were localized to the cell surface. Some rigorous washing steps improved the identification of all proteins, including those associated with the cell surface by three- to four-fold. Specifically, the eluted peptides were reintroduced onto the filter to perform multiple wash and re-elution cycles in 8 M urea buffer [35].

While FASP has demonstrated effectiveness in removing SDS, it is important to recognize that proteins are lost in the process [37–39]. This is partly due to incomplete digestion caused by extensive protein aggregation after removing SDS. To tackle this issue, Erde et al. [40] proposed the eFASP method, replacing the 8 M urea buffer with 0.2% SDC while pre-conditioning the filter with Tween-20. This modification led to improved trypsin digestion for both cytosolic and MPs and a three-fold reduction in peptide loss [40]. However, an independent evaluation by Nel et al. [41] found no significant difference in trypsin activity as both methods yield essentially the same number of MPs. In parallel, Ni et al. [42] introduced a modified FASP (mFASP) utilizing 0.4% SDC to enhance trypsin digestion. The mFASP approach involved purifying peptides over polymeric RP spin columns to aid in detergent removal. This modification not only improved the identification of hydrophilic peptides but also enhanced the identification of membrane-associated proteins [42]. In another approach, FASP was combined with a lectin-based affinity purification to isolate N-glycosylated proteins, a common characteristic of PMPs [43]. Following FASP, the eluted peptides were reintroduced onto the filter and incubated with lectins. Peptides were then eluted with PNGase F [43]. Deeb et al. [44] applied this approach to B-cell lymphoma cell lines (DLBCL). They identified 1304 unique N-glycoproteins, including 76% MPs of which 27% were PMPs. This method was sensitive enough to distinguish proteome differences among DLBCL subtypes [44]. Subsequently, Han et al. [45] applied the method to BV-2 mouse microglia, identifying 760 proteins, with 75% being MPs and 32% of them being PMPs.

Altogether, the FASP workflow has demonstrated its effectiveness. However, the method may not be applicable to detergents forming large micelles or having low CMC. DDM, for example, whose micelle size is over 70 kDa, will concentrate on the 10–30 kDa filter, while those with low CMC will require extensive washes [46]. These multiple centrifugation steps restrict its applicability in high-throughput processes [30,47] (Table 1). FASP's efficiency also decreases when working with small amounts of starting material (<10 µg), which is problematic for low abundance proteins like MPs [48]. Additionally, the release of formaldehyde from certain filters can lead to the modification of side chains leading to reduced protein identification [47,49]. Carbamylation can also occur on free amines due to the high concentration of urea in the FASP protocol [50]. These limitations should be considered when developing the FASP protocol in MP research and analysis.

**Table 1.** Extraction method comparison: time, material input, cost and throughput

	Time for 6–12 samples (h)	Minimum input material (µg)	Cost per sample	High throughput compatibility	References
FASP	∼4	10	$5	û	[28,31,48]
SP3	∼3	0.1	$1	✓	[28,48,52]
S-Trap	∼1	0.075	$10	✓	[28,59]

## Single-pot solid-phase-enhanced sample preparation (SP3) and SP3-solvent precipitation (SP4)

The SP3 technique utilizes carboxylate-modified magnetic beads (CMMBs) so that the entire process occurs in the same tube [51,52]. In a typical workflow, reduced and alkylated proteins are solvated in a water-miscible organic solvent, acetonitrile or ethanol, to prompt binding to the beads via hydrophilic interactions with the carboxylate group. Magnetic isolation of the beads then enables easier removal of detergents and contaminants. Protein digestion and elution occur in the same tube, minimizing sample loss and maximizing efficiency [51,52] (Figure 1). Since the SP3 protocol does not require centrifugation time, the process can be scaled up using magnetic racks, rendering it suitable for high-throughput applications [47] (Table 1).

In the context of membrane proteomic, SP3 has been combined with a cell surface capture procedure (CSC; intact cells are incubated with biocytin hydrazide so that glycoproteins can be isolated with streptavidin beads) to identify 600–900 surface glycosylated from 25 to 200 μg HeLa cell protein extracts [53,54]. This approach was also applied to CT26 colorectal cancer cells, using either cell culture or tumor xenografts, resulting in the identification of 900–1300 cell glycoproteins [53]. The method was reported sensitive enough to measure the relative protein expression between datasets [53]. Furthermore, Yan et al. [55] utilized CSC in conjunction with cysteine labeling to isolate cysteine-containing PMPs in Jurkat and primary T cells. Using SP3, they identified 1980 cysteine sites over 700 PMPs, including reduction-sensitive cysteine residues that affect the localization and uptake of low-density lipoprotein particles [55].

In a follow-up optimization, Batth et al. [56] reported the surprising observation that altering the surface chemistry of the CMMBs beads did not impact protein binding. They proposed that SP3 rather operates through protein precipitation driven by large aggregates that pellets upon solvation in organic solvents [57]. Organic solvents readily denature proteins, leading to strong interactions that drive aggregation. Contaminants such as SDS, however, typically remain in solution and thus can be removed [57]. Building on this finding, Johnston et al. [58] introduced SP4, a method utilizing inert glass beads to enhance the binding surface for the precipitated proteins. This approach, very cost-effective, also outperformed SP3 at higher protein concentrations, particularly for proteins with low solubility and high hydrophobicity. For instance, starting with the same sample, 192 proteins exhibited enhanced recovery in SP4 compared with SP3, and 47% of them were MPs. The authors proposed that glass beads may bind hydrophobic proteins more effectively than CMMBs or peptides elution from these beads is incomplete, especially when hydrophobic [58]. All in all, SP4 shows promise for MP identification, but due to its recent introduction, research findings are scarce.

## Suspension trapping

The S-Trap method, pioneered by Zougman et al. [59], employs a quartz or borosilicate glass filter placed over a RP membrane. The process begins by mixing reduced, alkylated, and solubilized proteins with phosphoric acid and a methanolic buffer (S-Trap buffer) to form larger protein aggregates. These aggregates are captured on the upper filter using spin-centrifugation or pressure-assisted devices. Subsequent washes with the S-Trap buffer removes contaminants, including detergents. Following digestion, peptides are no longer held by the glass filter and move towards the RP membrane for cleanup, fractionation if needed, and elution [59] (Figure 1). Notably, the S-Trap method is compatible with various detergent buffers although the use of SDS was found to be optimal [60].

The utility of the S-Trap method has been showcased in MP analyses. Zougman et al. [59] utilized the phase separation property of Triton X-114 to enhance MP isolation before applying S-Trap to remove the detergent. This approach identified over 3000 proteins in HeLa cells, with ∼45% belonging to the MPs category [59]. Building on this success, Chhuon et al. [61] validated the effectiveness of S-Trap for T cell lipid raft analysis, using it to eliminate iodixanol, an MS-incompatible reagent commonly employed for raft isolation via density gradient centrifugation. As a result, 2680 proteins, including 894 seemingly specific to the rafts were identified. Remarkably, methods such as FASP, in-solution and in-gel digestion were reported to be less effective than S-Trap in removing iodixanol [61].

Given the large pore size of the filter, S-Trap enables short centrifugation times [47] (Table 1), and commercially available 96-well plates further augments its utility, allowing for better reproducibility and high throughput workflow when dealing with a large number of samples [59]. However, it should be acknowledged that this efficiency comes at a significant cost, estimated at ∼$10 per sample, which is significantly higher than the other methods [47].

## Comparative analysis of FASP, SP3, SP4 and S-Trap

A limited number of evaluations have been conducted to compare the identification capabilities of these techniques. So far, the findings put some emphasis on the importance of material quantities when determining the most suitable preparation method. In Sielaff et al. [48] and Varnavides et al. [47], it was noted that both FASP and SP3 exhibited similar performance when starting with larger amounts of protein samples (>20 μg). However, at lower concentrations (<10 μg), SP3 outperformed FASP, showing a clear advantage of magnetic beads [48]. The performance of the methods was also influenced by the nature of the sample being analyzed. A study comparison using mouse microbiota revealed that S-Trap is superior in identification rates and quantification reproducibility compared with FASP and SP3 [62]. Similarly, when applied to colorectal cancer SW480 cells, the S-Trap method outperformed FASP in protein coverage [63]. However, when analyzing the bacteria *Klebsiella pneumoniae*, both S-Trap and FASP exhibited comparable identification and quantification [64]. Finally, Araújo et al. [65] demonstrated that FASP had higher protein identification in turbot liver tissues, whereas the S-Trap method excelled for mussel hepatopancreas. The method SP3, on the other hand, performed the second best for turbot samples and last for mussel samples [65]. These discrepancies in performance underscore the influence of sample nature on method efficacy.

Collectively, these nuanced observations highlight the need for systematic evaluations and standardized protocols to accurately assess the capabilities of the methods. Specifically, Zacchi et al. [66] demonstrated that S-Trap had superior efficacy in removing polymeric surfactants compared with SP3 and FASP, emphasizing the pivotal role of contaminant elimination in downstream processing and peptide recovery. Hayoun et al. [67] further illustrate this point by showing that S-Trap outperformed SP3 with shorter trypsin digestions, while SP3 surpassed it at higher digestion times, underscoring the complexity of these interactions. In the context of membrane proteomic, Yang et al. [68] reported an enhanced protein coverage for MPs and PMPs using the S-Trap method as opposed to FASP in milk fat globule membrane samples. Johnston et al. [58] noted similar performance in transmembrane protein recovery for SP4 and S-Trap. These findings underscore the absence of a definitive superior method. Crucial factors such as sample size, type, digestion duration, and contaminant removal all play determining factors, and it appears that each method should be fine-tuned to align the experimental setup.

## Membrane mimetics in proteomics

Despite the continual technological advances, MPs remain challenging for proteomic analysis, and more generally biochemistry and structural biology. To help manipulation, researchers have engineered membrane mimics such as nanodiscs, peptidiscs, and SMALPs. These membrane mimetics consist of an amphipathic scaffold that coats the hydrophobic parts of MPs, allowing them to exist in a water-soluble state without the need for detergents [69]. Consequently, these systems minimize the risk of protein aggregation, providing a more stable environment for downstream analysis [69]. They also aid with the removal of soluble protein contaminants, offering a promising solution to the inherent complexities of identifying and quantifying MPs in the proteomic workflow.

**Figure 2.**
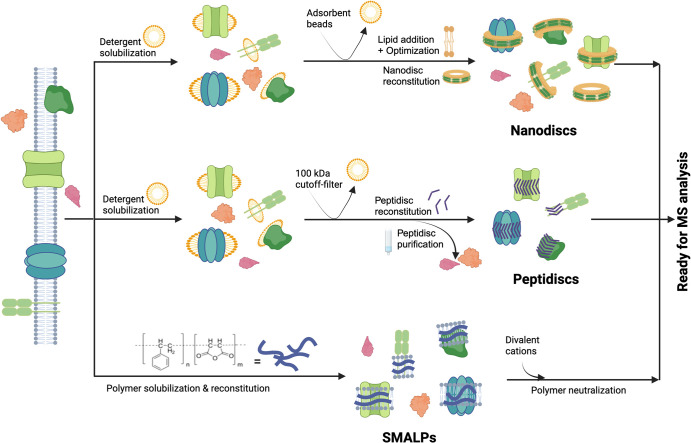
Workflows for membrane mimetic reconstitution of membrane proteins for bottom-up proteomics. This schematic illustrates the membrane protein extraction methods using Nanodisc, Peptidisc and SMALPs. Membrane proteins are captured in nanodiscs with exogenous lipids after detergent removal using adsorbent beads. Proteins are surrounded by lipids and encircled by membrane scaffold proteins (MSPs). In the peptidisc method, membrane proteins are captured without exogenous lipids and detergent removal occurs using centrifugation filters. Membrane proteins are surrounded by multiple amphipathic peptides and affinity tags directly on the peptides enable the isolation of the membrane proteome. In SMALPs, membrane proteins are extracted directly from the membrane. The SMA polymer wraps around membrane proteins and excess polymer must be removed prior to LC–MS/MS.

Nanodiscs, originally described by Bayburt et al. [70], are composed of a discoidal lipid bilayer encircled by a belt of two amphipathic proteins, derived from human ApoA1 protein, known as membrane scaffold proteins (MSPs). The reconstitution process involves mixing detergent-solubilized proteins with phospholipids and MSPs, followed by detergent removal using adsorbent beads to initiate the self-assembly process (Figure 2) [70]. In proteomic application, Roy et al. [71] first isolated MPs from human osteosarcoma 143B cell line using a two-phase (PEG and dextran polymers) separation system. The hydrophobic proteins, isolated in one layer, were solubilized with a nonionic glucoside detergent and eventually mixed with lipids and MSPs for reconstitution. The type of lipid added during reconstitution was found to influence protein recovery; for instance, GPCRs had better reconstitution in the presence of cholesterol, while cholesterol decreased the incorporation efficiency of other MPs [71]. The method was also applied to *Escherichia coli*, and here also not all MPs were incorporated in the nanodisc when using one set of conditions only [72]. Parameters such as MSP diameter, detergent removal process, type of lipids, and lipid-to-protein ratio were suspected to impact the reconstitution efficiency of the nanodisc [73,74], hence hindering its application to membrane proteomic.

The challenges posed by the MSPs were addressed using short amphipathic peptides known as peptidiscs [75]. Peptidiscs have demonstrated their ability to stabilize MPs of varying sizes without requiring additional lipids, thus simplifying the reconstitution process [75–78]. In the proteomic application, MPs were reconstituted in peptidiscs using 100 kDa-cutoff centrifugation filters that eliminate detergents while retaining the MPs in so-called peptidisc libraries (Figure 2). Notably, modifications to the peptidisc scaffold with his- or biotin-tags were introduced to allow library purification away from cytosolic protein contaminants [77]. The method was developed by Carlson et al. [76] and refined by Young et al. [77–79], who successfully isolated 1204 proteins from *E. coli*, with 49% of them being MPs. This method was further validated on HeLa and Panc-1 cells, underscoring its applicability for membrane proteome comparison [80]. More recently, the peptidisc approach was extended to five mouse organs including the fibrotic liver, demonstrating its ability to estimate the relative abundance of a range of PMPs and thereby the organ membrane proteome signature during disease development [81,82]. In all these studies, the detergent removal step was upstream MPs isolation, therefore reduction, alkylation, and trypsin digestion occurred all in the same tube, minimizing the risk of peptide loss. These advantages position the peptidisc as a promising approach in MP research. However, the peptidisc library construction demands a substantial amount of starting material, between 20 and 40 million cells [80]. Furthermore, the peptidisc peptides were overly abundant and overshadowed specific areas of the mass spectrogram, leading to incomplete protein identification [80].

A third membrane mimetic with potential application in membrane proteomic is the styrene-maleic acid polymer (SMA). This reagent enables direct membrane extraction of MPs within polymer-coated lipid particles, termed SMALPs (Figure 2). It is therefore an entirely detergent-free approach [83]. However, similar to ionic detergents, the SMA polymer carries a high negative charge density, which must be neutralized before MS analysis [84]. In a study by Morrison et al., the authors added divalent cations to destabilize and precipitate the polymer. Using 3T3L1 fibroblasts, they identified 205 integral MPs, but this number was lower compared with a classic detergent-based approach, perhaps due to incomplete removal of the polymer [83]. Efforts to reduce the charge of the polymer are going on. For instance, DIBMA substitutes aliphatic diisobutylene to aromatic styrene [84]. In a study by Scherhag et al. [85], membranes from *Pseudomonas aeruginosa* were solubilized with this novel polymer, followed by recovery of proteins using an acetone-based precipitation. This approach identified 3358 proteins, including 1194 MPs. However, similar to SMALP, the reagent was not so efficient in MP solubilization compared with the standard DDM detergent [85]. Challenges related to interference with analytical techniques and lower solubilization efficiencies highlight the need for further optimization and innovation in the field.

## Conclusion

Achieving an optimal balance between effectively solubilizing MPs with detergents while concurrently removing these reagents to prevent interference and aggregation remains a significant challenge in proteomics research. This dilemma raises a Shakespearean's question of whether to use detergents and risk interference with protein identification or to forgo detergents altogether, resulting in protein aggregation and loss. The methods described in this review have been developed to address this difficult issue and each offers unique advantages and limitations. As of today, the absence of a unique gold standard approach illustrates the ongoing complexity of this task and the need for further research and innovation in this area. We note that FASP, SP3/SP4 and S-Trap tend to dominate the bottom-up proteomic field, yet other relevant detergent removal strategies exist. Cleavable surfactants, column-based methods, phase-transfer, dialysis and electrophoresis techniques are continuously developed but were not expanded on in this review.

Some evaluation studies have been performed, but contradictions among them highlight the inherent challenges of this field. It becomes apparent that rather than direct method-to-method comparisons, the implementation of benchmarking experiments using well-characterized samples with standardized quantities may prove to be more beneficial. Such a benchmarked approach would help to recognize that a technique might seem efficient or sensitive when compared with another but also may miss important MPs or quantify them incorrectly. This is particularly important given that, beyond identification, an accurate quantification of MPs is essential for in-depth cellular analysis. The current evaluations rather focus on how many proteins are identified but often overlook the interfering background contaminants. Given that minimizing detergents, soluble proteins and protein aggregates is imperative for reliable and accurate results, assessing the portion of the spectral space occupied by these contaminants in comparison with MPs would yield a more precise evaluation of each method. An evaluation based on the method's ability to eliminate interference and comparing it to a benchmark experiment would contribute significantly to the comprehensive assessment of each technique.

Looking ahead, membrane mimetics show promise in overcoming challenges associated with detergents in proteomics. Unlike FASP, SP3, SP4 and S-Trap, membrane mimetics offer the advantage of swiftly removing detergents upon solubilization while preserving MPs in a water-soluble state. This feature reduces the risk of protein aggregation during downstream processes, addressing a concern that has not been effectively tackled by other detergent removal strategies. Nonetheless, membrane mimetics have limitations, as their co-elution can obscure MP signals. Implementing additional steps like chromatography (size-exclusion or ion exchange) or electrolysis before LC–MS/MS could be beneficial. Immunoprecipitation of membrane mimetics prior to LC–MS/MS may also prevent co-elution. Overcoming these challenges could offer a novel approach in bottom-up membrane proteomics, pending further evaluation to confirm unbiased protein identification. While membrane mimetics present a promising avenue, FASP, SP3/SP4 and S-Trap among others have demonstrated their effectiveness in capturing MPs as well.

## Perspectives

- Despite the ongoing technical challenge, the cell surface remains as a critical portal into the cell's interior, mirroring internal cellular processes. With a role in cell communication, responding to signals and transporting cargo, understanding MP content and variation allows researchers to uncover fundamental aspects of cell behavior. Going beyond identification, quantifying the cell membrane proteome facilitates comparisons, thus aiding downstream applications that define physiological and pathological conditions.
- Bottom-up proteomics addresses the challenges, yet the issue of MP sample preparation persists as a substantial limitation. A range of methods, such as FASP, SP3, S-Trap, and more recently membrane mimetics, have surfaced to tackle the complexities associated with detergents. Despite their effectiveness in various experimental setups, there is no one-size-fits-all solution or singular optimal approach. The choice depends on numerous factors, including sample types, quantity, cost, efficiency, type of detergents, and the intended output to be measured. As the field continues to advance, the ongoing development and assessment of these diverse methods remain crucial to refining sample preparation for bottom-up proteomics studies.
- One-on-one comparisons are being conducted to evaluate different methods, but future comprehensive approaches should also compare each method against carefully chosen benchmarks. This benchmarking would enable a direct assessment of quantification accuracy, surpassing mere identification capabilities. Membrane mimetics holds promise as they skip detergents in downstream analysis while preventing MP aggregation simultaneously — an integration not achieved by other methods. Future research validating their capacity, or developing new mimetics or methods that unbiasedly capture the membrane proteome would be a major advance in the field.

## Open Access

Open access for this article was enabled by the participation of University of British Columbia in an all-inclusive *Read & Publish* agreement with Portland Press and the Biochemical Society under a transformative agreement with Individual.

## Abbreviations

CMC: critical micelle concentration
CMMB: carboxylate-modified magnetic bead
FASP: filter-aided sample preparation
mFASP: modified FASP
MP: membrane protein
MSP: membrane scaffold protein
PMP: plasma MP
RP: reverse-phase
SMA: styrene-maleic acid
